# Triplex DNA Helix Sensor Based on Reduced Graphene Oxide and Electrodeposited Gold Nanoparticles for Sensitive Lead(II) Detection

**DOI:** 10.3390/toxics11090795

**Published:** 2023-09-20

**Authors:** Jing Gao, Piao Xu, Lu Qiao, Yani Tao, Yao Xiao, Hong Qin, Yuan Zhu, Yi Zhang

**Affiliations:** 1 College of Environmental Science and Engineering, Hunan University, Changsha 410082, China; gaojing@hnu.edu.cn (J.G.); piaoxu@hnu.edu.cn (P.X.); qiaolu@hnu.edu.cn (L.Q.); taoyani@hnu.edu.cn (Y.T.); xiaoyao@hnu.edu.cn (Y.X.); qinhong@hnu.edu.cn (H.Q.); 2 Key Laboratory of Environmental Biology and Pollution Control, Ministry of Education, Hunan University, Changsha 410082, China

**Keywords:** DNA sensor, triple helix, T-A•T, G-quadruplex, Pb^2+^ detection

## Abstract

A triplex DNA electrochemical sensor based on reduced graphene oxide (rGO) and electrodeposited gold nanoparticles (EAu) was simply fabricated for Pb^2+^ detection. The glass carbon electrode (GCE) sequentially electrodeposited with rGO and EAu was further modified with a triplex DNA helix that consisted of a guanine (G)-rich circle and a stem of triplex helix based on T-A•T base triplets. With the existence of Pb^2+^, the DNA configuration which was formed via the Watson–Crick and Hoogsteen base pairings was split and transformed into a G-quadruplex. An adequate electrochemical response signal was provided by the signal indicator methylene blue (MB). The proposed sensor demonstrated a linear relationship between the differential pulse voltammetry (DPV) peak currents and the logarithm of Pb^2+^ concentrations from 0.01 to 100.00 μM with a detection limit of 0.36 nM. The proposed sensor was also tested with tap water, river and medical wastewater samples with qualified recovery and accuracy and represented a promising method for Pb^2+^ detection.

## 1. Introduction

Lead, as one of the most widely utilized metal materials, is not biodegradable and can cause serious health and environmental issues [1,2]. Therefore, it makes a lot of sense to precisely and sensitively detect and analyze it in the fields of environment monitoring, agricultural industry, food tracking and clinical toxicology et al. [3,4]. Thus far, traditional detection methods, such as inductively coupled plasma mass spectrometry (ICP-MS) [5,6], high-performance liquid chromatography (DHPLC) [7,8] and cold vapor atomic fluorescence spectrometry (CVAFS) [9,10] have been utilized for lead detection. These detection technologies can accurately, stably and sensitively determine targets. However, the large equipment of these detection technologies is difficult and costly to maintain, and complex to operate. Also, with the increasingly complex pollutant compositions and the smaller order of pollutant concentrations causing low damages, it is difficult to provide enough appropriate methods for the requirements of analyses and determinations now [11,12]. Nevertheless, the sensitive, rapid and low-cost sensors applied for target detections have gained more attention, and offer a potential method for online and in situ monitoring. So far, many researchers have employed diverse biosensing methods for Pb^2+^ detection and monitoring. Pb^2+^ biosensors could be constructed with a portable and paper distance device based on specific DNA capture with rapid detection performance in real water samples [13]. A biosensor based on a spherical nucleic acid fluorescence probe binding on Au nanoparticles was fabricated for the specifical detection of Pb^2+^ in complex samples due to its excellent nuclease resistance [14].

Nucleic acids, as receptors in electrochemical sensors, can specifically react with targets and cause configurational changes. So far, abundant and diverse DNA configurations have been developed for sensor fabrications, such as metal-mediated DNA, triple helix DNA construction and G-quadruplex [15,16]. There are two typical metal-mediated DNA which have been used to develop heavy metal biosensors, CC mismatches mediated by Ag^+^ and TT mismatches mediated by Hg^2+^. A fluorescent biosensor was developed based on C-Ag^+^-C base pairs for Ag^+^ detection [17]. In addition, Hg^2+^ could be detected through dual-mode fluorescence and colorimetric probes of T-modified AuNPs/reduced-state sulfur nanodots because of the special bonding of T-Hg^2+^-T in water samples [18]. Triple Helix, as a late-start DNA configuration, can also be applied for DNA sensor fabrications. It is composed of a DNA duplex and a single strand (ssDNA). The sources of ssDNA are the strand itself with a folding or foreign strand. This ssDNA can combine with the duplex via Hoogsteen binding. The Hoogsteen pairs possess quite different properties to Watson–Crick base pairs [19,20]. The formation of Watson–Crick base pairs depends on hydrogen bond force. Nonetheless, Hoogsteen base pairs provide the N7 positions of purine bases (as hydrogen bond acceptors) and C6 amino groups (as donors), which combine on the Watson–Crick (N3-C4) face of the pyrimidine base [21,22]. There are two types of base pairings of Hoogsteen, purine–pyrimidine–purine and pyrimidine–purine–pyrimidine. Also, the G-quadruplex strategy is another DNA configuration for sensor construction. It is a nucleotide sequence with a conjunction structure that consists of G-rich bases. It has been found that positive ions, especially Pb^2+^, can promote stability after embedding into the cavity of G-quadruplex [23]. The hydrogen bond is the main binding force in the duplex. However, besides the hydrogen bond, other forces of the interaction between molecules control the configuration of more advanced DNA [24]. Therefore, Pb^2+^ has sufficient ability to break the triplex helix and form a more stable G-quadruplex. Hence, the probe of triplex DNA helix obtained a G-rich circle and a stem of triplex helix with T-A•T base triplets was adopted for the development of the Pb^2+^ sensor. The signal transduction was caused by the configurational change of the DNA probe when Pb^2+^ was sensitively and selectively captured by the triplex DNA helix and G-quadruplex formed.

The promotion of recognition and transduction is a significant research trend for the development of high-performance sensors based on the characteristics of appropriate nanomaterials. Until now, various nanomaterials have been employed for sensor fabrication [25,26]. Owing to its prominent physicochemical characteristics, graphene has been in the spotlight of scientific research. It has the properties of high specific surface area and charge carrier mobility, and low resistivity that is suited for sensor fabrication. The development and economization of non-silicon materials, especially graphene, have been some of the main areas of research in sensing method practical applications in real environments [27,28]. However, the property of hydrophobicity restricts the utilization of graphene in sensors to a certain extent. Conversely, the electrical conductivity of graphene oxide (GO) is low, though it is rich in oxygen-containing groups [29]. In order to combine the advantages of two nanomaterials, rGO is synthesized. Comparing the methods of chemical vapor deposition and graphene function, electrodeposition is a more convenient method [30]. GO can be directly reduced on substrates, which avoids the unevenness of rGO on the substrates and reduction in electrical conductivity. As a result, rGO was applied in our research. Gold nanoparticles (GNPs) are also excellent nanomaterials for sensor developing. They are easily synthesized and have a high surface-to-volume ratio and low electrical resistivity [31]. In addition, the favorably biocompatible GNPs are able to provide a suitable platform for biological molecules [32]. Moreover, GNPs can also be rapidly and directly electrodeposited on electrodes without chemical reagents to maintain electrical conductivity as far as possible. In addition, the use of NPSs avoids a series of chemical changes and reactions and the complicated controlling of the reaction conditions.

Therefore, a simple DNA electrochemical sensor based on a probe containing a G-rich circle and a stem of triplex DNA helix with T-A•T base triplets was proposed. The configuration of that probe was broken with triplex helix splitting and G-quadruplex forming with Pb^2+^. MB, which had an affinity for G embedded into G-quadruplex, acted as the signal indicator. It is demonstrated that the sensor can realize the sensitivity and selectivity for Pb^2+^ detection. Furthermore, the Pb^2+^ sensor used in tap water, Xiangjiang River water and medical wastewater samples in order to test its practicability in real water.

## 2. Materials and Methods

### 2.1. Materials and Apparatus

Mercury nitrate (Hg(II)), methylene blue (MB), tris(2-carboxyethyl)phosphine (TCEP) and mercaptohexanol (MCH) were acquired from Sigma-Aldrich Chemical Co. (Shanghai, China). Hydrogen tetrachloroaurate trihydrate (HAuCl_4_·3H_2_O, 99.9%) and other chemicals were bought from Sinopharm Chemical Reagent Co., Ltd. (Shanghai, China). The appliance buffers and solutions in this work were as follows: the DNA stock buffer was prepared with 10 mM Tris-HCl which contained 0.1 mM TCEP, 1 mM EDTA and 1.0 M KCl, pH 8.0; the supporting electrolyte solution was blended with 10 mM Tris-HCl which contained 10 mM KCl, pH 7.4; the washing buffer was prepared with 10 mM PBS which contained 0.1 M NaCl, pH 7.4; and the redox couple solution was prepared with 5 mM [Fe(CN)_6_]^3−/4−^ (1:1 M ratio) which contained 0.1 M KCl. Ultrapure water (18.2 MΩ/cm) was employed in all operations. Sangon Biotech. Co., Ltd. (Shanghai, China) synthesized the oligonucleotides probe, and the sequence was S1: 5′-SH-(CH_2_)_6_-CTTCTGGGATGGGCGGGATGGGTCTTCAGAAG-3′. It was preserved at −20 °C for utilization.

Electrochemical measurements were operated with the CHI760B electrochemistry system produced by Chenhua Instrument (Shanghai, China). The three-electrode system consisted of a GCE as the working electrode (WE), a saturated calomel electrode (SCE) as the reference electrode and a Pt foil counter electrode. A JSM-6360LV scanning electron microscope (JEOL Ltd., Tokyo, Japan) was utilized for Scanning Electron Microscopy (SEM). Transmission electron microscopy (TEM) images were created with a JEOL-1230 electron microscope (JEOL, Japan) at 100 kV. The syntheses of rGO were carried using cyclic voltammetry (CV), and the potential ranged from −1.5 to 0.5 V with a sweep speed of 0.01 V/s. Chronoamperometry was applied for EAu syntheses in the potential of 0.18 V. DPV was performed in the potential changing from −0.8 to 0 V under 25 mV amplitude, 4 mV pulse width and 0.2 s pulse period.

### 2.2. Sensor Fabrication

The GCE was polished with 0.25 μm and 0.05 μm alumina powder on suede for 30 min in sequence, washed with acetone, absolute alcohol and ultrapure water and then dried. The three-electrode system was immersed into 0.5 M H_2_SO_4_ solution for CV scanning. The potential ranged from −1.0 to 1.0 V with a sweep speed of 0.1 V/s for removing the impurities on the surface of GCE. Then, GCE was washed and dried after the CV curves completely overlapped.

A total of 1 mg GO was dispersed into 1 mL PBS solution (pH = 9.18). In order to obtain thin GO nanosheets, the GO solution underwent ultrasonic treatment for 30 min. The cleaned GCE was immersed into the treated GO solution for CV scanning for 10 cycles with nitrogen gas injected at 0 °C. RGO was reduced and modified on the GCE. The three-electrode system was continuously immersed into 5 mL 1% (wt) HAuCl_4_ solution containing 0.5 M perchloric acid for the electrodeposition of EAu for 50 s, and then EAu/rGO/GCE was fabricated.

S1 was reacted in a polymerase chain reaction (PCR) machine for 5 min from 90 °C down to room temperature in order to form a triplex helix. A total of 1.0 μM S1 with 30 μL was dropped on the surface layer of EAu/rGO-modified GCE overnight at 4 °C. MCH was covered on S1/EAu/rGO/GCE for 30 min.

### 2.3. Detection

The S1/EAu/rGO/GCE was immersed in various concentrations of Pb^2+^ solution for 30 min at 25 °C. Then, the modified electrode was immersed in a 10 mM Tris-HCl buffer with 20 μM MB and 1.0 M KCl (pH 7.4) and left to stand for 15 min. After soaking in the Tris-HCl buffer without MB for 15 min, the DPV was performed for Pb^2+^ detection.

## 3. Results and Discussion

### 3.1. Design of the Sensor Strategy

**Scheme 1:**
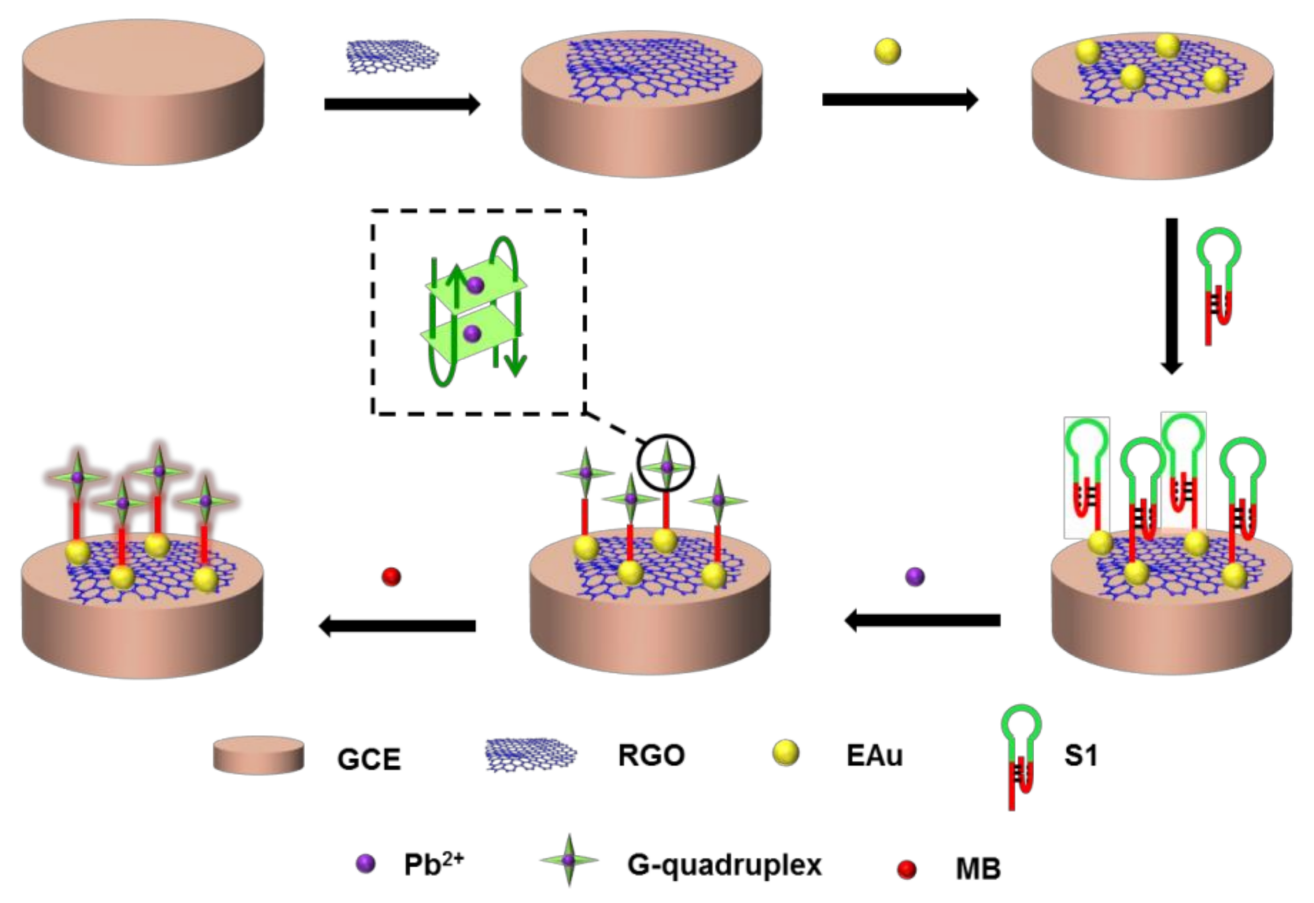
Scheme of the DNA sensor for Pb^2+^ detection.

Regarding the sensor strategy, shown in Scheme 1, GCE covered by rGO and EAu successively was modified with S1 via a Au−S bond. The triplex DNA helix split and formed a more stable G-quadruplex in Pb^2+^ solution. MB entered the G-quadruplex for signal indication. The configuration changes between the triplex DNA helix and G-quadruplex with Pb^2+^ capturing could be detected by the sensor with the electrical response. The signal transduction was related to the variation in DNA configurations that occurred only upon binding. S1, as the receptor, allowed for sensitive and selective capturing of Pb^2+^, even in a complex environment with changeable and complicated compounds. The sensor would exhibit a satisfactory detection performance in theory.

### 3.2. Characteristics of Electrode

**Figure 1:**
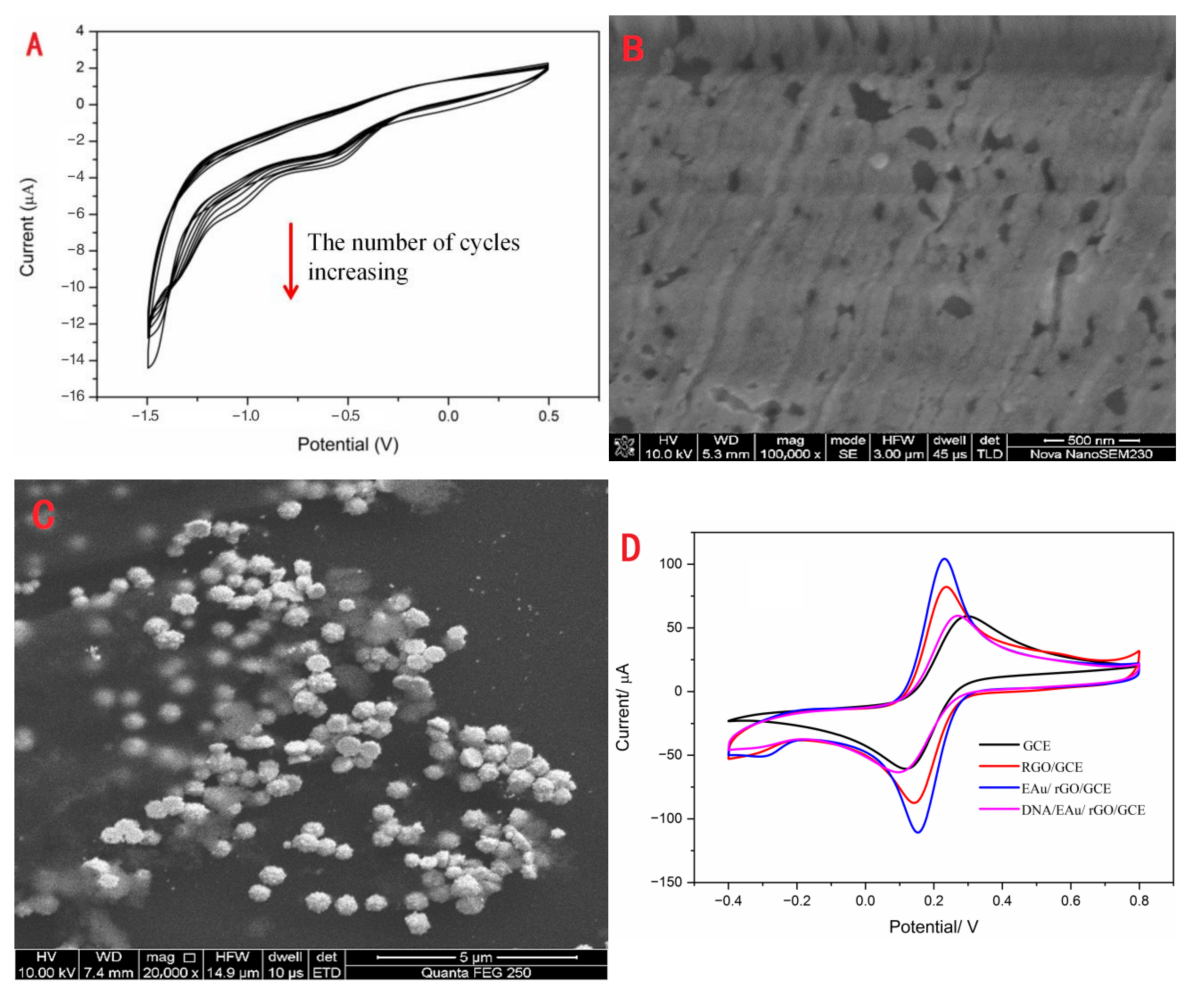
The characteristics of the modification materials: the CV (**A**) and SEM (**B**) of electrodeposition of rGO, the SEM of EAu/rGO (**C**), and CV (**D**) of bare GCE, rGO/GCE, EAu/rGO/GCE and DNA/EAu/rGO/GCE.

RGO and EAu were covered on the surface of GCE in sequence using the electrodeposition method. The cyclic voltammogram of rGO is exhibited in Figure 1A. The voltammetric current obviously increased during the process of successive potential scanning. This illustrated that rGO was successfully electrodeposited on GCE. Also, the SEM image (Figure 1B) of rGO/GCE could demonstrate that the rGO layer covered the GCE as a film. The grooves of the rGO layer were the scratches of GCE. As seen in Figure 1C, the EAu was successfully electrodeposited on the rGO/GCE with a particle size of about 0.65 μM. The electrochemical performances of electrodes modified with different nanomaterials are exhibited in Figure 1D. The bare electrode possessed the minimum difference redox peak current. After rGO and EAu coating on the GCE, the peak current responses obviously increased, and the peak potential decreased. This indicated that these nanomaterials could efficiently promote electron transfer. With the combination of S1, the peak current sharply decreased because the DNA was non-conductive biomolecular. This demonstrated that S1 was tightly combined on the modification electrode.

### 3.3. Optimization of the Experimental Conditions

For achieving the optimum current signal response, a sequence of condition tests was carried out by DPV. The parameter analyses of S1/EAu/rGO/GCE were executed with three replicates. All the optimization experiment parameters and experiment steps were followed as shown in Section 2.2 via the controlling variable method. The experimental conditions effect of pH, electrodeposition time of EAu, treatment time of MB, and reaction time between S1 and Pb^2+^ ions were tested under the condition of 0.5 μM Pb^2+^.

**Figure 2:**
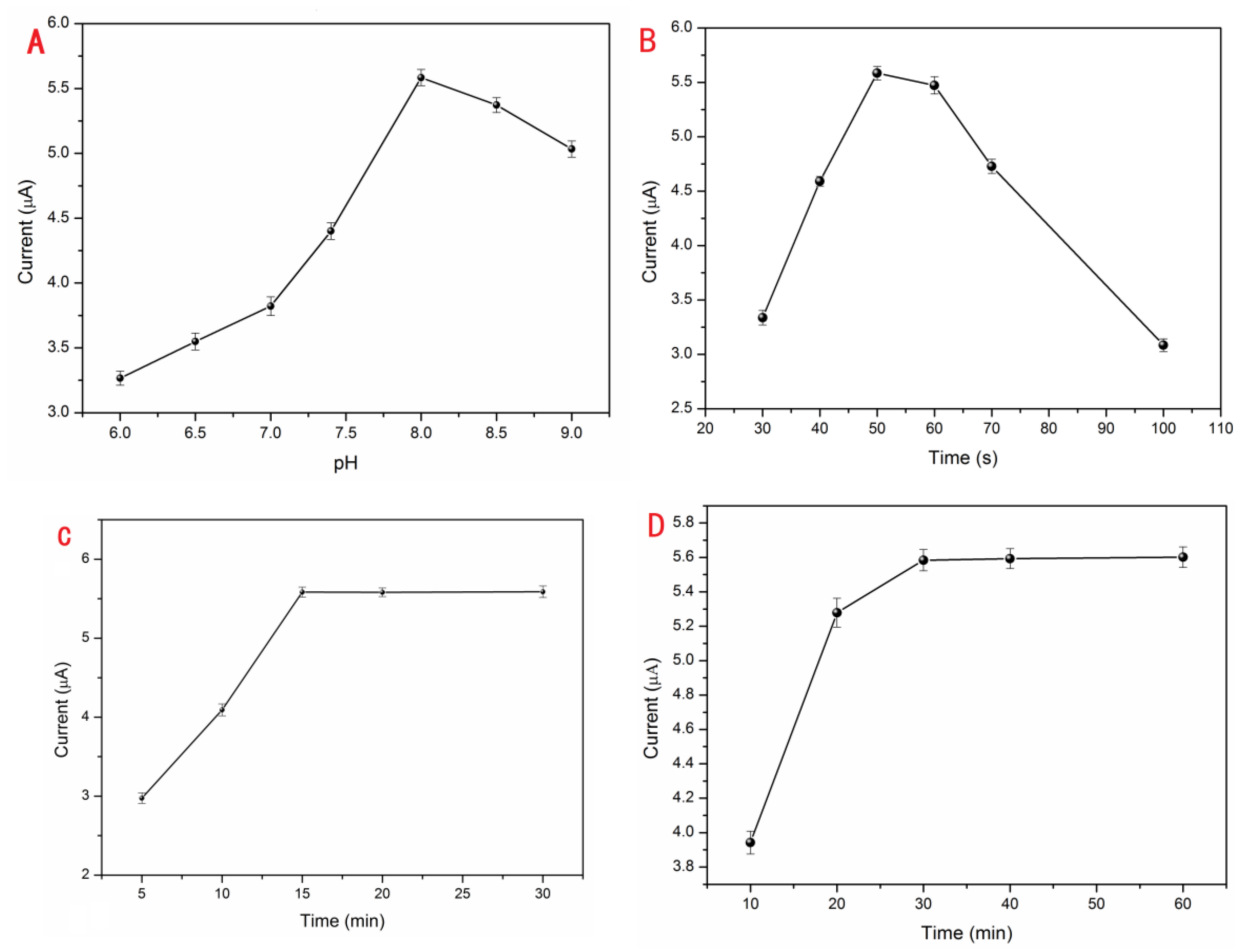
The optimization of experimental conditions: the effects of pH (**A**), electrodeposition time of EAu (**B**), treatment time of MB (**C**), Pb^2+^ reaction time (**D**).

The GCE modified by S1/EAu/rGO was run in different pH values of 10 mM Tris buffer containing 0.5 μM Pb^2+^ and 10 mM KCl. In Figure 2A, the peak current responses obviously increased in the pH range from 6.0 to 8.0, and then they slowly decreased from 8.0 to 9.0. The greatest peak current response was at the pH value of 8.0. The triplex DNA can reversibly deform in various pH solutions [33]. It possesses high stability in acidic solution, and the bridge of T-A•T can be separated in a basic solution. The DNA probes easily reacted with Pb^2+^ ions and transformed into a G-quadruplex at pH 8.0 with the increasing current. But, the current weakened in high pH basic solution. So, the optimal pH 8.0 condition was used in the subsequent experiments. For the purpose of further improving electrode performance and providing more binding sites for DNA probes, EAu were rapidly electrodeposited on the surface of rGO/GCE to obtain the maximum peak current response (Figure 2B). After the electrodeposition of rGO and EAu, the electrode was continuously fabricated, as described in Section 2.2. The optimal quality for EAu could be beneficial to the EAu/rGO system and effectively improve the detection performance of the proposed Pb^2+^ sensor. An inadequate amount of EAu could not provide enough reaction sites for DNA probe binding. So, the current signal was enhanced with electrodeposition time increasing, and the current reached its peak in 50 s. Meanwhile, the current responses decreased when the electrodeposition time exceeded 50 s. The reason for this is that the effect of rGO can be weakened which makes it resemble the effect of a bare Au electrode with an excessive amount of EAu. Moreover, the rGO/GCE was coated with a thick layer of EAu which restricted the electronic transmission in rGO. After modification with S1/EAu/rGO, the electrode was immersed into 20 μM MB and 1.0 M KCl (pH 7.4) for MB loading and then DPV-tested in 0.5 μM Pb^2+^ Tris buffer. As the current signal of the proposed sensor, the capacity of MB could directly affect the current signal. The fabricated electrode was immersed in MB solution for 15 min with a remarkable peak current response. There were almost no current changes even if it was treated with MB for over 15 min (Figure 2C). Therefore, a treatment of 15 min with MB was adopted in subsequently executed tests. In the test for the optimum reaction time among DNA and Pb^2+^ ions (Figure 2D), the S1/EAu/rGO electrode was treated with MB for 15 min, then tested with the 0.5 μM Pb^2+^ DPV current. The response current was promoted, and achieved maximum at the time of 30 min, which indicated the split and hybridization reactions with Pb^2+^ were completed. Meanwhile, the response current barely changed. Therefore, the condition of 30 min was elected as the optimum reaction time for capturing Pb^2+^.

### 3.4. Detection of Pb^2+^

**Figure 3:**
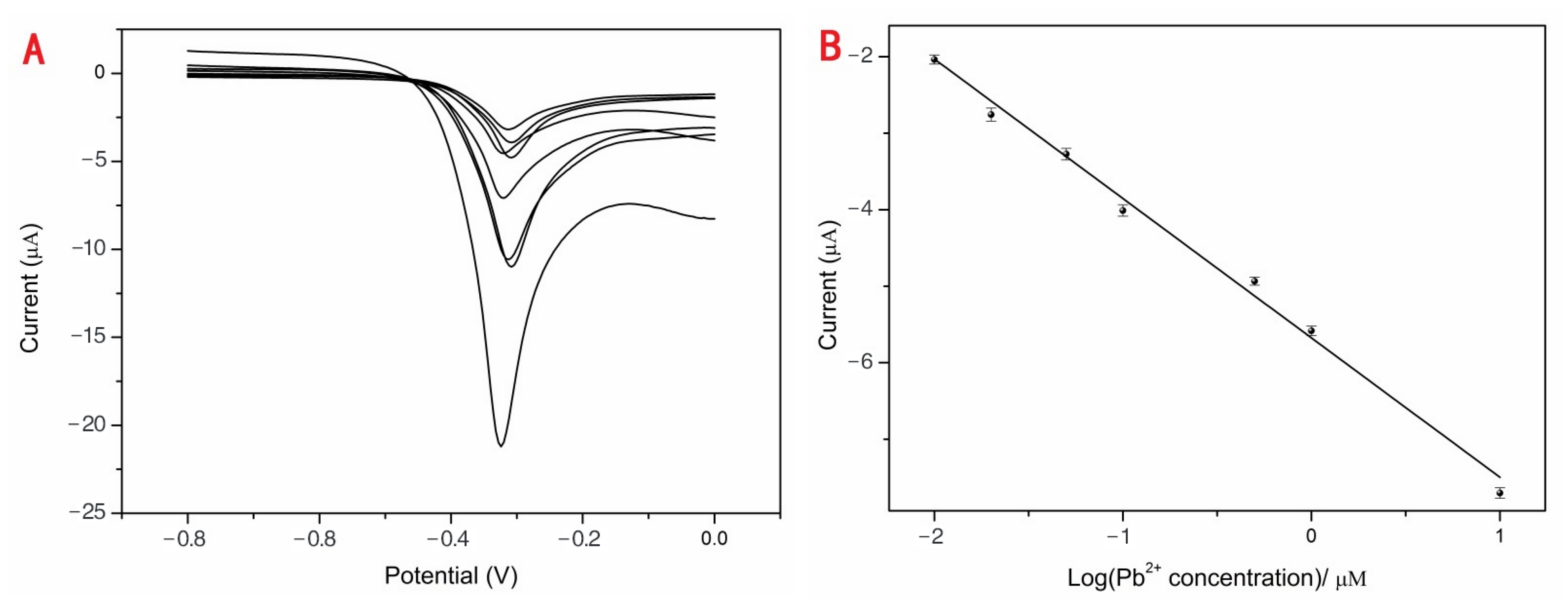
The DPV responses of Pb^2+^ sensor (**A**). The current responses increased with concentrations of Pb^2+^: 0.01, 0.02, 0.05, 0.10, 0.50, 1.00, 10.00 and 100.00 μM. The linear relationship between the DPV peak current changes and the logarithm of concentrations of Pb^2+^ (**B**).

Various concentrations of Pb^2+^ (0.01, 0.02, 0.05, 0.10, 0.50, 1.00, 10.00 and 100.00 μM) from one stock solution were investigated using DPV with the optimum conditions to estimate the performance of the developed sensor. The blank group data were deducted, as shown in Figure 3A. With the increasing Pb^2+^ concentrations, the current responses were obviously enhanced. The linear detection range from 0.01 to 100.00 μM was achieved between the peak currents and the concentrations of Pb^2+^ (Figure 3B). The correlation equation was (1) with a corresponding regression coefficient (R^2^) of 0.9919. The average current value for 0 nM Pb^2+^ was 0.0257 μA (n = 10). Original values of different Pb^2+^ concentrations were subtracted from the background current value of 0.0257 μA and the data presented in Figure 3A,B. The standard deviation of 0 nM Pb^2+^ (n = 10) is 0.00068, and S was the slope of the calibration obtained from the correlation Equation (1) 5.6762. The *σ* was the standard deviation (SD) of a blank solution (n = 10) and S was the slope of the calibration. The detection limit of the proposed Pb^2+^ sensor was 0.36 nM (3*σ*/S). The drinking water LOD criterion of Pb^2+^ is 0.01 mg/mL (about 48.3 nM) according to GB5749-2006 of China’s “Sanitary Standards for Drinking Water” [34]. The proposed sensor is the highest permissible rank of Pb^2+^ in drinking water following detection.

Y = (−5.68 ± 0.08) C − (1.82 ± 0.07),(1)

The points of calibration were carried out in triplicate, and the precision of the sensor was guaranteed. The highly sensitive Pb^2+^ sensor could easily achieve the detection and recognition of Pb^2+^ meeting the maximum allowable concentration of Pb^2+^ in drinking water. We are currently analyzing the different types of DNA sensors for Pb^2+^ detection, (Table 1), such as colorimetry with a limit detection of 35.25 pM [34], surface-enhanced resonance Raman scattering (SERS) with a limit detection of 3.55 pM [35], fluorescence with the limit detection of 5.7 nM [36] et al. The high detection performance of the proposed sensor could be ascribed to the electrical conductivity improved by EAu/rGO and the specific probe for Pb^2+^ capturing.

### 3.5. Selectivity of the Sensor

**Figure 4:**
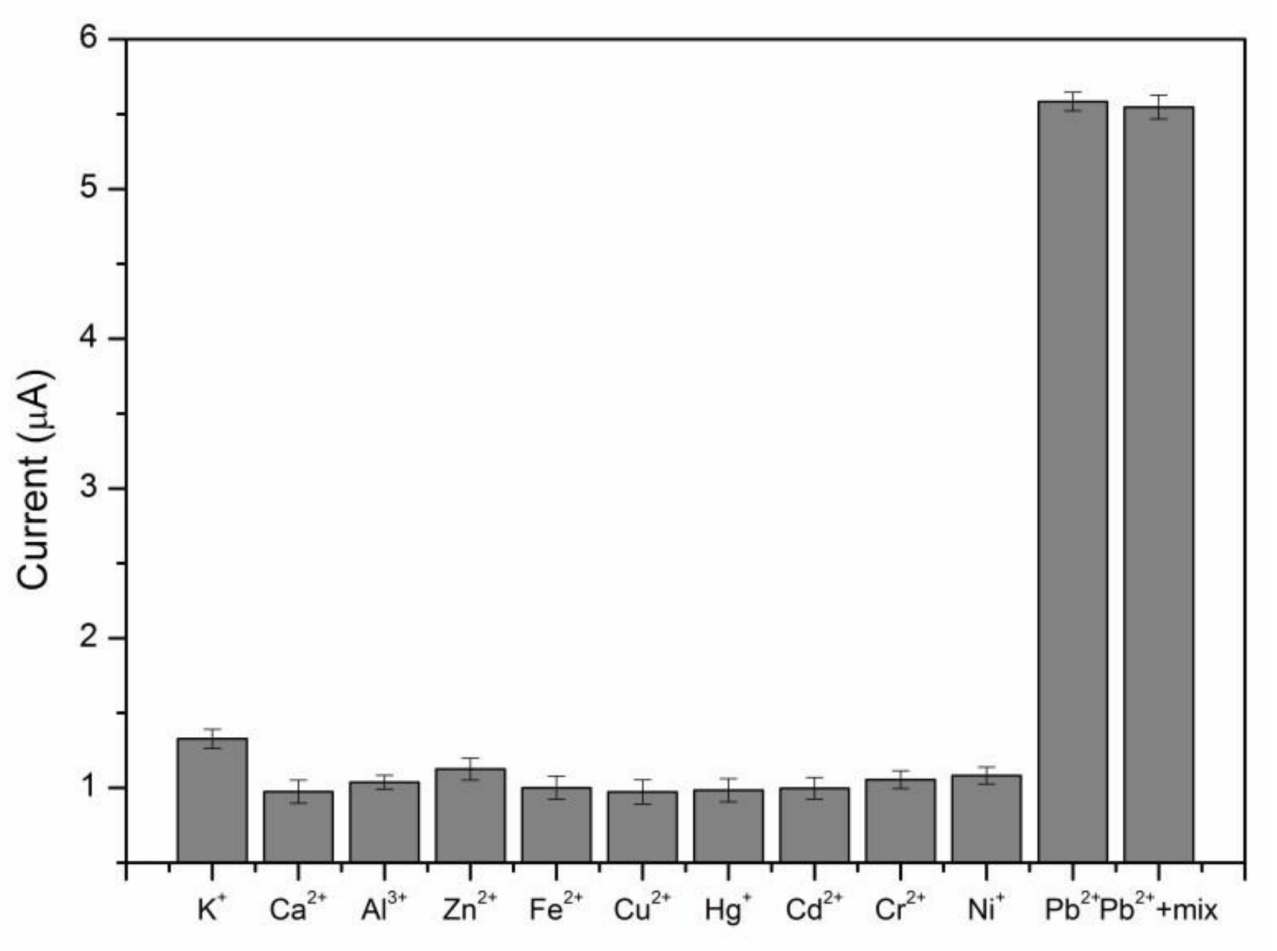
The selectivity of the electrochemical sensor for Pb^2+^. The concentrations of Pb^2+^ and other metal ions were 0.5 μM.

To evaluate the selectivity of the proposed sensor, several potential interference metal ions, such as K^+^, Ca^2+^, Al^3+^, Zn^2+^, Fe^3+^, Cu^2+^, Hg^2+^, Cd^2+^, Cr^2+^, Ni^+^ at a concentration of 0.5 μM, and their mixtures with the optimum conditions were studied (Figure 4). The peak current responses for these closely relevant metal ions with the environment were not enough to be interfered with compared with those of 0.5 μM Pb^2+^. The result illustrated that the proposed sensor exhibited excellent selectivity for Pb^2+^.

### 3.6. Analysis of Pb^2+^ in Samples

The Pb^2+^ sensor was further tested in real water samples. These waters were sampled from tap water, Xiangjiang River water and medical wastewater, and then were briefly filtered by the centrifuge and spiked with Pb^2+^. The samples were collected from our laboratory tap water, the Pailou Road section of Xiangjiang River and medical wastewater of Hunan University Hospital, 1 L each, in July. The samples were available. Tap water with industrial purification is close to our lives and research. River water is not only close to our lives and research, but is also typically a real water in our surrounding environment with unpredictability and complication. Medical wastewater samples contained various organic compounds. They were used for detection after filtration and sterilization. In order to compare the detection results, the three kinds of real samples, with preprocessing, were detected by the developed Pb^2+^ sensor (Table 2). The recoveries of Pb ^2+^ in real water samples were in the range from 93.7% to 101.0%, which demonstrated that the Pb^2+^ sensor had great potential for practical detection in a real water environment.

### 3.7. Reproducibility and Stability of Sensor

The reproducibility of the developed sensor was investigated. In order to remove the DNA probe, the working electrode was immersed in Piranha solution for 30 min after each measurement, then washed and dried for further DNA probe modification and a 0.5 μM Pb^2+^ detection test in optimal conditions. A relative standard deviation (RSD) value of 4.7% was obtained for five successive detections. The repeatability of the Pb^2+^ sensor based on S1/EAu/rGO/GCE was also tested in 0.5 μM Pb^2+^ solution with the same fabrication and detection steps under optimal conditions. A RSD value of 3.9% was obtained from five different GCEs. This indicated that the fabrication procedure was reliable and the sensor possessed good reproducibility and repeatability.

## 4. Conclusions

A triplex DNA helix electrochemical sensor fabricated with rGO and EAu for Pb^2+^ detection was simply developed. The rGO and EAu directly electrodeposited on GCE, which avoided certain cumbersome works and saved time compared with large-scale and sophisticated detection instruments. Pb^2+^ could be specifically recognized by the triplex DNA helix which consisted of a G-rich circle and a stem of triplex helix based on T-A•T base triplets for the formation of G-quadruplex. The proposed sensor demonstrated a linear relationship between the DPV peak current responses and the logarithm of Pb^2+^ concentrations from 0.01 to 10 μM with a limit detection of 0.36 nM. The sensor was also tested with tap water, Xiangjiang River water and medical wastewater samples with good recovery and accuracy, representing a promising approach for Pb^2+^ detection. It was confirmed that the rGO and EAu with excellent physicochemical properties were suitable for sensor development. The designed DNA probe possessed good selectivity and sensitivity for Pb^2+^, and it was a potential biomaterial for Pb^2+^ capture in Pb^2+^ detection and contaminated remediation. Also, we can continuously simplify the preparation steps, develop more stable and reliable nanomaterial systems and combine them with current commercial portable devices and technologies, such as personal glucose meters and mobile phones, for further practical application in real-water environments.

The proposed Pb^2+^ sensor possessed good selectivity and sensitivity, but there were still limitations or challenges encountered during the research. The sensor production process contained many steps and it almost took one day. In order to ensure the detection performance of the sensor, the conditions of fabrication were strictly controlled, such as rGO electrodeposition and DNA reaction temperatures, reaction times, et al. The sensor was carried in alkaline solution with good performance. Real-world water environments are unpredictable and complicated, as we know. This impacts detection performance. Also, the activity of the DNA probe affected the regeneration of the sensor. These limitations and challenges in our research present the greatest challenges for sensors in practical application. Globally, researchers have been trying to overcome the above-mentioned difficulties.

## Figures and Tables

**Table 1:** Comparison of different Pb^2+^ detection methods.

Target	Detection Limitation	Detection Range	Method	Ref.
Pb^2+^	35.25 pM	0.1–200 nM	Colorimetry	[35]
	3.55 pM	10 nM–100 μM	SERS	[36]
	5.7 nM	25–1400 nM	Fluorescence	[37]
	6.1 nM	0.01–10 μM	Fluorescence resonance energy transfer (FERT)	[38]
	2.61 pM	1 pM–100 μM	Electrochemistry	[12]
	0.36 nM	0.01–100 μM	Electrochemistry	This work

**Table 2:** Pb^2+^ concentrations in real samples determined by the sensor.

Samples	Spiked Pb^2+^ (μM)	Senor (μM) ^1^	Recovery (%)
Tap water	0.100	0.101 ± 0.07	101.0%
Xiangjiang River water	1.000	0.937 ± 0.06	93.7%
Medical wastewater	0.100	0.100 ± 0.08	100.0%

^1^ Mean values for triplicate experiments ± standard deviation.

## Data Availability

The data underlying this article will be shared upon reasonable request to the corresponding authors.
